# Development and Nerve Supply of the Intestinal Canal Surgically Considered

**Published:** 1910-08

**Authors:** J. L. Campbell

**Affiliations:** Atlanta, Ga.; Professor Surgical Anatomy and Clinical Surgery Atlanta School of Medicine; Visiting Surgeon Wesley Memorial Hospital and Tabernacle Infirmary


### DEVELOPMENT AND NERVE SUPPLY OF THE INTESTINAL CANAL SURGICALLY CONSIDERED.

BY J. L. CAMPBELL, M. D., ATLANTA, GA.

*Professor Surgical Anatomy and Clinical Surgery Atlanta School of Medicine; Visiting Surgeon Wesley Memorial Hospital and Tabernacle Infirmary.*

As the development of the digestive system has been well understood for a number of years, there is nothing new that can be said about it, but a review of old things is sometimes of interest.

A short time after the fertilization of the ovum the cells begin to differentiate and fold off from the embryonic area. Three layers are formed. The external one, the ectoderm forms the external covering of the body. The internal layer, the entoderm, forms the epithelial lining of the intestinal canal and all the glands developed from it. while the middle layer, the mesoderm, forms the peritoneum, the walls and frame work of the body and digestive organs, but has nothing to do with glandular function.

The mesoderm divides into two layers, a parietal and visceral. The former more or less closely connected with the ectoderm, forms the somatopleure. The latter is similarly connected with the entoderm to form the splanchnopleure. The somatopleure folds off from the embryonic area in four directions; an anterior or cephalic, a posterior or caudal and two lateral folds, the four finally meeting at a point which later becomes the umbilicus. These folds encolse a large cavity, the coelom or body cavity, which is divided into the peritoneal, pericardial and pleural cavities.

The splanchnopleure folds downward forming a tube—the primitive intestinal canal, which at this time is straight, of uniform thickness and closed at both ends and near its middle it communicates with the yolk sac by means of a tube, the vitelline or vitello-intestinal duct. That part of the primitive intestinal canal contained within the cephalic fold is known as the foregut. From it are developed the salivary, thymus and thyorid glands, the pharynx, oesophagus, stomach, two-thirds of the duodenum, the gall bladder. liver, pancreas and all the glands in the mucous membrane of this part of the intestinal tract. That portion contained within the lateral folds is known as the midgut—from it are developed the small intestines. caecum, appendix, ascending and transverse colon to the splenic flexure. The portion contained in the caudal fold is known as the hindgut—from it are developed the splenic flexure, descending colon, sigmoid flexure and rectum, except the anal orifice.

The primitive intestinal canal is suspended from the dorsal wall of the embryo within the coelom by a fold of mesoderm, which later becomes the mesentory.

The first evidence of the stomach is seen as an enlargement, which occurs within the first three or four weeks—a little caudad to the middle of the foregut. It is suspended in a fold of mesoderm extending from the dorsal to ventral wall to the coelom. This fold is known as the meso-gastrium. It is limited cephalad by the septum transversum from which the diaphragm is developed. Caudad the ventral portion of the meso-gastrium, has a free margin in a part of which the umbilical vein runs to the liver. This later forms the free margin of the falciform ligament of the liver while the remainder forms the free border of the gastro-hepatic omentum.

The liver and gall bladder are developed by an evagination of the endomeric cells of the duodenum, pushing into the ventral mesogastrium, which forms its peritoneal covering for four of the ligaments of the liver and the gastro-hepatic omentom.

A few days after this the pancreas begins to develop by a similar budding into the dorsal mesentery of the duodenum. At first there are two portions, one passing directly dorsal, the other a little ventral and nearly parallel to the intestinal tube. Later as the duodenal loop forms these fuse, the horizontal portion forming the head, and the perpendicular part, the body and tail of the adult pancreas.

At first the stomach is symmetrical in shape and lies parallel to the long axis of the body. The dorsal border grows much more rapidly than the ventral. This causes a sagging of the cephalic or cardiac end. An elevation of the caudal or pyloric end takes place, probably due to shortening of the ventral mesogastrium by the development of the liver.

The growth of the dorsal border—the future greater curvature in excess of the ventral border, the future lesser curvature and the drawing of the pyloric end cephalad causes the stomach to rotate to the right. The liver also rotates in the same direction and becomes adherent to the dorsal wall of the coelom and the back part of the caudal surface of the diaphragm. Thus the right side of the stomach becomes the dorsal and the left side the ventral surface of the adult stomach. By this rotation the dorsal mesogastrium having increased in length is carried from left to right over the pancreas and a bursa or pouch is formed below the greater curvature. It continues to increase in length as development proceeds and falls over the transverse colon. The two layers thus brought into contact fuse and later become attached to the transverse colon. It continues to increase in length and passes caudad and ventrad to the small intestines, forming the great omentum.

Caudad to the stomach a short or duodenal loop is formed in the intestine consisting of the caudal end of the foregut, and the cephalic end of the midgut. By the sixth week the duodenal loop has assumed a transverse position below the ploric end of the stomach. This is brought about the rotation already described. The lower three-fourths of the loop, the right leaf of its mesentery, and the right surface of the pancreas fall against the dorsal wall of the coelon and the two layers of peritoneum thus brought into contact fuse and the left leaf of the primitive mesentery forms the dorsal parietal peritoneum. Thus the greater part of the duodenum is extra peritoneal.

Caudad to the duodenal loop a second or umbelical loop is formed, consisting of the remainder of the midgust which has developed in length rapidly and is carried ventrad into the umbilical vesicle outside the abdominal cavity by the contraction of the vitelline duct which is attached by one end to the apex of the loop and by the other to the apex of the umbilical visicle; in this way the so-called "umbilical or embryonal intestinal hernia" is formed.

In its simplest form the loop consists of a descending or proximal and an ascending or distal limb which develops respectively into the jejunum and ileum, the caecum and appendix, ascending and transverse colons. The proximal end of the loop forms the duodeno-jejunal angle, the distal end of the splenic flexure of the colon. The loop is attached to the dorsal abdominal wall by an elliptical shaped mesentery down the middle of which the omphalo-mesenteric (the future superior mesentery) artery passes. Dorsad the two extremities of the loop approach each other forming the duodeno-colic isthmus.

Differentiation of the large from the small intestine is marked as early as the sixth week by the caecal outgrowth which takes place on the convex surface of the ascending limb of the umbilical loop a short distance from the attachment of the vitelline duct. Caudad to this the intestine grows slowly in length but more rapidly in size. Very early the caecal bud shows an unequal rate of development, the distal portion growing slowly forms the appendix, while the proximal portion develops into the caecum proper. The development of the caecum can be divided into five stages: (1st) a circumscribed thickening of the coats of the canal. (2nd) a sac-like enlargement on the convex side of the ascending limb of the umbilical loop. (3rd) the sac assumes a crescentic shape with the concavity to the right. (4th) the crescent becomes funnel shaped. (5th) a further constriction occurs at the neck of the funnel and the appendix is formed, while a further sacculation takes place above and the future variations in shape of the caecum depend on this change.

Cephalad to the caecum the development is more rapid in length and definite convolutions are formed even before its withdrawal from the umbilical vesical, which takes place about the tenth week when the vitelline duct is obliterated. Occasional remains of this duct are seen in the adult as Meckel's diverticulum. Immediately after its withdrawal convolutions begin to form rapidly. The jejunal convolutions occupy the left upper, the upper iliac, the right lower quadrants and the lower iliac the caudal portion of the cavity. The caecum, which up to this time, has been on the left lower side of the cavity and intestinal coils is carried cephalad along the median line until it reaches almost to the liver and stomach and the whole canal begins to rotate on the superior mesenteric artery as an axis at the duodeno-colic isthmus. The caecum is carried from beneath the stomach across the descending portion of the duodenum. The lower iliac coils of the intestines are still dorsad to it and the concavity of the caecum is still directed to the right, but as it reaches the caudad surface of the liver ventrad to the right kidney the coils of intestines rotate to the left, the caecum rotates on its axis through a half circle so that concavity points to the left and the ileocaecae junction assumes the adult form. From this point the caecum passes caudad along the right side of the cavity until it reaches the adult position in the right iliac fossa.

The primitive intestinal canal distal to the duodeno-colic isthmus is pushed to the left side of the cavity and the left leaf of the mesentery falls against the dorsal wall and is attached so that the right leaf becomes the dorsal parietal peritoneum. Thus the splenic flexure and descending colon have no mesentery, while the sigmoid flexure in its course from right to left retain a short mesentery. The rectum is pushed against the dorsal wall of the pelvis and the mesentery is obliterated, except at the extreme proximal end.

We have now briefly traced the changes from the primitive straight tube of the four weeks embryo to the complicated digestive system of the adult.

The divisions of the primitive intestinal canal have each a distinct function to perform and have each a distinct or nearly so, nerve supply, while all the abdominal viscera are supplied by the vagi and sympathetic nerves, yet the divisions are well marked.

The stomach, duodenum, gall bladder, liver and pancreas, are closely related, all being developed from the foregut are supplied by the vagi, branches from the right phrenic and the hepatic plexus of the sympathetic. The right phrenic is distributed more to the peritoneum than to the muscles and mucous portions of the organ.

The jejunum, ileum, caecum, appendix and colon, to the splenic flexure, which are developed from the midgut, are supplied through the superior mesentery plexus. The splenic flexure, descending colon, sigmoid and rectum by branches from the inferior mesentery plexus.

There are three systems of nerves in the abdominal wall. (a) nerves to the skin; (b) nerves to the muscles; (c) nerves to the parietal peritoneum; any one or all of these three sets may be the seat of the referred pain and soreness, the most common being the muscular nerves, this pain is erroneously regarded as being situated in the diseased viscus." (Treves applied Anatomy.)

Dr. Head has shown that each abdominal viscus has a definite connection with sensory areas in the cerebro-spinal system and thus accounts for the reflex pains felt in the thoracic and abdominal walls. He says that "When a painful stimulus is applied to a part of low sensibility in close central connection with a part of much greater responsibility the pain produced is felt in the part of higher sensibility rather than in the part of lower sensibility to which the stimulus was actually applied." (Quoted from Howell's Phys.) Mr. Treves says: "The brain being accustomed to localize pain only along the spinal nerves makes a mistake and refers the pain along the spinal nerves of the segment disturbed." (Treves' Applied Anatomy.)

It is impossible to trace with accuracy, the definite connection between the nerve fibres supplying a viscus and the cerebro-spinal nerve with which they connect, because of the different plexuses through which they pass.

This paper is written with especial reference to reflex symptoms of diseases of the gall bladder and appendix, while other portions of the intestinal canal furnish as many reflexes we cannot study them in so short a time. Diseases of each of these two organs are accompanied not only by well-marked reflex symptoms of pain and tenderness over certain portions of the abdominal and throracic walls, but by disturbances of functions in allied organs. This latter is probably dependent as much on embryological relation as on the nerve supply.

Many patients suffering from cholecystitis have their stomach treated for hyper-acidity and their backs blistered for rheumatism, while the real cause goes untouched. "The pain in cholecystitis may vary from mere discomfort to great severity." (a) The majority of cases suffer with pain in the right hypochondriac region and along the right costal margin. (b) Nearly all suffer with pain in the right side of the epigastric region. (c) Forty to fifty per cent. of the cases suffer with pain and tenderness at the lower angle of the right scapula and tenderness along the course of the eighth and ninth thoracic nerves, more marked where the cutaneous branches are given off. (d) There is frequently a tender spot three finger breadths internal to the acromion process on the anterior border of the trapezius muscle. Pain is sometimes felt at this point when pressure is made on the fundus of the gall bladder and pain may be produced in the gall bladder by pressure on this spot. This is most marked when there is peritonitis in the gall bladder region. (e) Rigidity of the abdominal muscles is persent when there is peritonitis.

In order to account for these reflex phenomena we must conclude that the sensory nerves supplying the gall bladder have a close central connection with the sensory nerves supplying the areas above mentioned. The eighth and ninth thoracic nerves communicate with the right great splanchnic which enters the semilunar gangila and the hepatic plexus and finally the gall bladder. The peritoneum of the gall bladder receives filaments from the right phrenic which is made up of branches from the fourth and fifth cervical nerves. These nerves also furnish the cutaneous branches for the area along the anterior border of the trapezius.

Pain and tenderness are frequently experienced from deep pressure over th appendix area. This may be producd by (1) pressing the abdominal contents against the gall bladder or (2) by the two organs being closely allied than has hitherto been suspected. In quite a large per cent. of my cases, I have found it necessary to remove the appendix at the same time that I have operated on the gall bladder.

"The gall bladder, liver, duodenum, pancreas and stomach, are embryologically, anatomically, physiologically and pathologically, closely related and should be considered a * * * * * physiological system," (MacCarty in Annals of Surgery). All being developed from the foregut, are concerned in the preparation of the food for absorption by the midgut—any diseased condition of one affects the others, and the food is sent into the absorbing portion of the canal unprepared, therefore the whole body suffers from lack of nourishment. Hence, a general debility in long standing cases of gall bladder disease.

The stomach is first affected by disease of the gall bladder, and so constant and persistent is the symptom of "indigestion" that it is often with great difficulty that the patient can be convinced that the lesion is not located in that organ. Certain foods seem to start an attack and the average patient cannot understand how indigestible food in one part of the digestive system can cause pain in another.

The pancreas and deodunum suffer next, for the proper secretion of the pancreas and duodenal glands depend on certain elements of stomach secretion. These failing to perform their functions causes the food to undergo putrifactive changes, and poison instead of nutrition is absorbed.

I have already said that the appendix is affected in a large number of cases of gall bladder disease. It was found to be involved in 13 per cent. of a series of 365 cases operated on at the Mayo Clinic. In my experience it has occurred more often. At any rate, disease of the two, are so often associated that it should always be examined and if certain reflex symptoms I am about to mention, be present, the appendix should be removed, unless the condition of the patient forbids it.

During the height of an acute attack of appendicitis the most constant referred pain is to the lower epigastric region. So marked is this, that have often seen applications applied to this part of the abdomen and the appendix area left entirely alone. Pressure on the appendix at this stage will often be followed by pain a little to the left of the median line in the above mentioned area, but it is not in acute attacks that the greatest value can be obtained from reflex pains.

Robert T. Morris has called attention to a second point that is of importance in chronic appendicitis, especially where interstitial changes are taking place and the nerve filaments are being caught in the scar tissue; this point is a little to the right of the umbilicus over the right lumbar ganglia of the sympathetic cord. When the appendix alone is involved, the left lumbar ganglia are never tender, but if the rectum or the tubes and ovaries or the uterus are involved, the left will be tnnder also. One is frequently able to diagnose a latent case of appendicitis by this means when there is no tenderness over McBurney's point.

The referred symptoms in respect to the interference with functions in other organs are much the same in this as in gall bladder diseases, though not so closely allied to the stomach embryologically, indigestion is one of the most common symptoms of chronic appendicitis, some even go so far as to assert that by interfering with the proper functions of the stomach and gall bladder appendicitis is a cause of gall stone.

Since Clado demonstrated the connection between the appendix and right ovary by means of the appendiculo-ovarion ligament and vessels, the influence of chronic appendicitis over the ovary has been well recognized. In quite a per centage of cases

I have found it necessary to remove the right tube and ovary at the same time that the appendix was removed.

In conclusion, I wish to emphasize the necessity of studying these reflex symptoms which can be understood only by a knowledge of the development and nerve supply of the intestinal canal and its connection with the cerebro-spinal nervous system.

References:

*Gray's Anatomy.*

*Pearsal's Anatomy.*

*Deaver's Surgical Anatomy.*

*Deaver & Ashhurst—Surgery of the Upper Abdomen. Vol. I.*

*Treve's Appiled Anatomy.*

*Howell's Physiology.*

*Huntington's Anatomy of the Peritoneum and Abdomen.*

*Francis Campbell—Surgical Anatomy.*

*Rudolph Schmidt's "Pain."*

*W. C. Macarty—Pathology of the Gall-Bladder and Some Associated Lesions—Annals of Surgery, May, 1910.*

*Robert T. Morris—McBurney's Point and Another Point in Appendix Diagnosis—J. A. M. A., Vol. I., No. 4.*

*Robert T. Morris—My Present Position on the Appendix Question.—J. A. M. A., Vol. LI. No. 8.*

*Brubaker's Text Book of Physiology.*

*F. P .Mall—Bulletin Johns Hopkins Hospital, Vol. IX. Nos. 90-91.*

*Cunningham's Anatomy.*

